# Item Response Theory Analyses of Diagnostic and Statistical Manual of Mental Disorders, Fifth Edition (DSM-5) Criteria Adapted to Screen Use Disorder: Exploratory Survey

**DOI:** 10.2196/31803

**Published:** 2022-07-27

**Authors:** Mathieu Boudard, Jean-Marc Alexandre, Charlotte Kervran, Louise Jakubiec, Dvora Shmulewitz, Deborah Hasin, Lucie Fournet, Christophe Rassis, Patrice Claverie, Fuschia Serre, Marc Auriacombe

**Affiliations:** 1 Sanpsy (Sleep Addiction and Neuropsychiatry), CNRS (Centre National de la Recherche Scientifique), UMR (Unité Mixte de Recherche) 6033 University of Bordeaux Bordeaux France; 2 Pôle Interétablissement d'Addictologie Centre Hospitalier Charles Perrens and Centre Hospitalier Universitaire de Bordeaux Bordeaux France; 3 MéRISP (Methods for population health intervention research), BPH (Bordeaux Population Health), INSERM (Institut National de la Santé et de la Recherche Médicale) Unité 1219, University of Bordeaux Bordeaux France; 4 Department of Psychiatry Columbia University New York, NY United States; 5 Mairie de Martignas Martignas France

**Keywords:** screen media use, screen addiction, internet gaming disorder, screen use disorder, Item Response Theory

## Abstract

**Background:**

Screen use is part of daily life worldwide and morbidity related to excess use of screens has been reported. Some use of screens in excess could indicate a screen use disorder (ScUD). An integrative approach to ScUD could better fit the polymodal reality of screens, and concurrent problems with screens, than a split approach, activity by activity. In that paradigm, a pragmatic and operationalized approach to study a potential ScUD requires the use of common criteria, for all screens and activities done on screens, in a single questionnaire.

**Objective:**

Our goals were (1) to describe screen uses in a general population sample and (2) to test the unidimensionality, local independence, and psychometric properties of the 9 Diagnostic and Statistical Manual of Mental Disorders, Fifth Edition (DSM-5) internet gaming disorder (IGD) criteria adapted to screen use in a community sample. We hypothesized that the 9 DSM-5 IGD criteria adapted to ScUD would show unidimensionality, local independence, and good discrimination, with criteria distributed on the severity continuum.

**Methods:**

This cross-sectional survey in a French suburban city targeted adults and adolescents. A self-administered questionnaire covered the main types of screens used and their use for various activities in the past month. Presence of ScUD diagnostic criteria in past 12 months was also self-evaluated in the questionnaire. Factor and 2-parameter Item Response Theory analysis were used to investigate the dimensionality, local independence, and psychometric properties of the ScUD criteria.

**Results:**

Among the 300 participants, 171 (57.0%) were female (mean age 27 years), 297 (99.0%) used screens, 134 (44.7%) reported at least one criterion (potential problem users), and 5 (1.7%) reported 5 or more criteria and endorsed an ScUD. The most endorsed criteria were loss of control (60/300, 20.0%) and preoccupation (52/300, 17.3%). Screen types used and screen activities differed between participants with no ScUD criteria and those with at least one ScUD criterion. The latter were more likely to have a computer as the most used screen type, and more video gaming, communication/social network, and watching news and research of information as activities. Unidimensionality was confirmed by all fit indices. Local independence was confirmed by the absence of residual correlation between the items. Criteria had relatively high factor loading, with loss of interest in other recreational activities having the highest. However, criteria with the lowest factor loading all remained above the cut-offs, sanctioning unidimensionality. Most discriminating criteria were loss of interests, preoccupation, deceive/cover up, and risk/lose relationship/opportunities, which also provided the most information on the measurement of the latent trait.

**Conclusions:**

We described screen uses in a French community sample and have shown that the adaptation of the DSM-5 IGD to “ScUD” has good psychometric validity and is discriminating, confirming our hypothesis. We suggest to use those criteria to assess potential “ScUD.” Further studies should determine if all criteria are needed and whether others should be added.

## Introduction

Increased affordability and functionality of screen devices have contributed to making screen use part of current daily life worldwide [1-4]. Screen use facilitates communication for leisure-related activities (ie, video games, social media) and access to knowledge for education and work-related activities. However, some adverse consequences of using electronic screens have been reported. Sleep [5-7], visual problems [6,8], and overweight and obesity [9] have been associated with screen use. Excessive screen use has also been associated with a drop in academic accomplishments [10], psychiatric disorders [11], and suicide in adolescents [12]. All of these are related to duration of use and could be the expression of a potential addiction to screens [13,14]. Although the link and the direction of the link between screen use and increased mortality and morbidity remain to be confirmed [15,16], there is enough evidence to explore whether such a screen use disorder (ScUD) could be diagnosed for the purpose of prevention and treatment.

Based on clinical similarities with addictions, and the significant damages related to video game use, the American Psychiatric Association (APA) included internet gaming disorder (IGD) in the third section of the *Diagnostic and Statistical Manual of Mental Disorders, Fifth Edition (DSM-5)* in expectation of further research [17,18]. The 9 IGD criteria were adapted from gambling disorder criteria, with a threshold of 5 to qualify for the diagnosis. Some criteria are common with those of substance use disorder. Differences are no craving or time spent criterion, an adverse negative mood, and a deceive/cover up criterion. Studies showed that IGD criteria have good psychometric validity with unidimensionality and good discrimination [19,20]. However, specific features of IGD are debated, including validity of the criteria and how to better operationally define them [18,21,22].

Many screen activities represent potentially addictive behaviors, and problematic media use has been studied on many screen types, such as “gaming disorder” [18,22,23], “smartphone use disorder” [24,25], and “internet addiction” [26]. Other authors have adapted IGD criteria to assess other potential behavioral addictions, such as “social media disorder” [27-29] and “screen media addiction” [14]. Considering clinical observations [30] and existing studies, we suggest combining these disorders into one “ScUD,” characterized by the DSM-5 IGD criteria adapted to screen use [31]. We do not imply that screens are of themselves addictive, but that the combination of screen portability with ongoing internet access reduces time from decision to action and to positive reinforcement, which increases the addictive potential [32] of activities mediated by screen use. Screens offer a much higher availability, even permanent, of not just 1 activity but all of them at the same time, on the same medium, for almost everyone. Besides, internet connection may potentialize them (in terms of incitation, salience, rewards, problems, etc.). From a nosographic perspective, the study of a potential disorder of screen use with an integrative approach could better fit the polymodal reality of screens, and concurrent problems with screens, than a split approach, activity by activity. In that paradigm, a pragmatic and operationalized approach to study a potential ScUD requires the use of common criteria, for all screens and activities done on screens, in a single questionnaire.

Item Response Theory (IRT) postulates that a latent construct or trait that is not directly observable such as the proposed ScUD can be measured by a group of criteria [33]. These are the preferred analyses for assessing dimensional and structural validity of diagnostic criteria, such as IGD or substance use disorder criteria [19,20,34-37]. In recent studies on IGD that included gamers recruited via gaming websites or social media [19,20], screen media “addiction” in parents’ reports of their children’s behavior [14] showed that the IGD criteria fit well with the 1-factor model and that some criteria were more discriminant than others. However, to our knowledge, no study has yet assessed IGD criteria adapted to screen use using IRT among general population samples.

In 2015, Martignas-sur-Jalle (Nouvelle-Aquitaine, France) city council requested a local survey about screen uses (n=7400). This was an opportunity to conduct a general population survey of the IGD criteria adapted to screen use. Our goals were, in a suburban community sample, (1) to describe screen use and (2) to test the unidimensionality, local independence, and psychometric properties in terms of difficulty and discrimination of the 9 DSM-5 IGD criteria adapted to screen use. We hypothesized that the 9 DSM-5 IGD criteria adapted to ScUD would show unidimensionality, local independence, and good discrimination, with criteria distributed on the severity continuum.

## Methods

### Study Design

We designed an exploratory survey among the population of Martignas-sur-Jalle (n=7400). A task force with the University of Bordeaux, Charles Perrens Hospital Addiction Clinic, Martignas-sur-Jalle city council, and population representatives was established to carry out and supervise the survey conducted from January 4, 2016, to February 25, 2016.

### Participants

The study targeted all adults and adolescents from middle-school age (ie, from 11 to 12 years of age) with no upper age limitation. The task force agreed on this minimal age to assess screen users and ensure understanding of the questions. There were no exclusion criteria.

### Procedure

**Figure 1:**
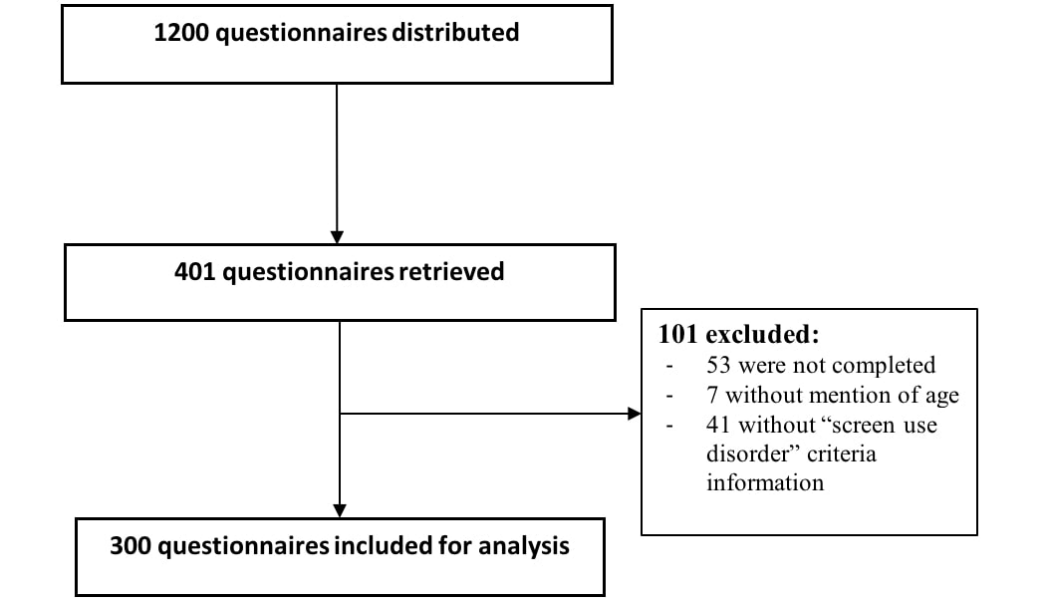
Flow chart of questionnaires selection process.

Participants received the questionnaire from distribution points (all city services and schools) and returned them directly through ballot boxes or mail. Of the 1200 questionnaires distributed, 401 were returned. The response rate was 33.4% and the sample represented 6.6% of the target population of the city. After a quality check, 101 questionnaires were excluded (53 with no information, 7 without age, and 41 with ScUD questions not completed). The remaining 300 questionnaires were used for the database (Figure 1).

### Survey Questionnaire

The survey questionnaire was a 10-15-minute 2-part self-administrated questionnaire including 49 closed-ended questions designed by the task force. The first part (37 questions) explored the main types of screens used over the past month (eg, TV, computers, smartphones, tablets, and handheld consoles) and for which activities (communication, social media, work, searching information on internet, other documentation, shopping, gaming, gambling, and others). The second part assessed each ScUD diagnostic criteria in the past 12 months (9 questions) and which screens and activities were considered problematic, that is, when at least one ScUD criterion was endorsed (1 question for screens and 1 for activities). We used the previously published French translation of the 9 IGD criteria [18] and adapted them to screen use (the term “videogames” was replaced by “screens”). The original French version and the translated version of the questionnaire are provided in Multimedia Appendix 1.

### Measures

Our variables of interest were sociodemographic data (age, sex), screen use (converted into hours per day), activities, main screen used, main activities, prevalence of each diagnostic criteria, and ScUD. Activities were quantified by the number of days in the past 30 days (participants had to choose out of the following 4 options: every day or almost every day; more than 1 day out of 2; less than 1 day out of 2; and never or almost never). The main screen used was defined as the prevalence of participants for whom this screen was the most used (frequency over the past 30 days multiplied by the time per day). The main activity was defined for each activity as the prevalence of participants endorsing the activity on the main screen. ScUD was defined when 5 or more criteria were reported on the scale adapted from IGD (Table 1).

**Table 1:** Screen use disorder criteria.

Criteria	Internet gaming disorder	Screen use disorder
Preoccupation	Do you spend a lot of time thinking about games even when you are not playing, or planning when you can play next?	Do you spend a lot of time thinking about screens, even when you are not using them, or planning when you can use them next?
Withdrawal	Do you feel restless, irritable, moody, angry, anxious, or sad when attempting to cut down or stop gaming, or when you are unable to play?	Do you feel restless, irritable, moody, angry, anxious, or sad when attempting to cut down or stop using screens, or when you are unable to use screens?
Tolerance	Do you feel the need to play for increasing amounts of time, play more exciting games, or use more powerful equipment to get the same amount of excitement you used to get?	Do you feel the need to use screens for increasing amounts of time, use more exciting screens, or use more powerful equipment to get the same amount of excitement you used to get?
Loss of control	Do you feel that you should play less, but are unable to cut back on the amount of time you spend playing games?	Do you feel that you should use less screens, but are unable to cut back on the amount of time you spend using screens?
Loss of interest	Do you lose interest in or reduce participation in other recreational activities (hobbies, meetings with friends) due to gaming?	Do you lose interest in or reduce participation in other recreational activities (hobbies, meetings with friends) due to screens?
Continue despite problems	Do you continue to play games even though you are aware of negative consequences, such as not getting enough sleep, being late to school/work, spending too much money, having arguments with others, or neglecting important duties?	Do you continue to use screens even though you are aware of negative consequences, such as not getting enough sleep, being late to school/work, spending too much money, having arguments with others, or neglecting important duties?
Deceive/cover up	Do you lie to family, friends, or others about how much you game, or try to keep your family or friends from knowing how much you game?	Do you lie to family, friends, or others about how much you use screens, or try to keep your family or friends from knowing how much you use screens?
Escape adverse mood	Do you game to escape from or forget about personal problems, or to relieve uncomfortable feelings such as guilt, anxiety, helplessness, or depression?	Do you use screens to escape from or forget about personal problems, or to relieve uncomfortable feelings such as guilt, anxiety, helplessness, or depression?
Risk/lose relationship/opportunities	Do you risk or lose significant relationships, or job, educational, or career opportunities because of gaming?	Do you risk or lose significant relationships, or job, educational, or career opportunities because of screen use?

### Statistical Analysis

#### Overview

We first described sociodemographic data. Quantitative variables were described by means and SD, and categorical variables with percentages. Adolescents and adults were analyzed together unless specified differently. Main activities and screen types for participants with no ScUD criteria versus those with at least one ScUD criteria were compared in univariate (Pearson tests) and multivariate analyses (logistic regression, controlled on age and gender). Statistical significance was set at *P*<.05. The prevalence of participants endorsing at least one ScUD criteria was compared between adults and teenagers. On an exploratory basis, participants with potential screen use problem (defined here as at least one criterion endorsed) were compared with those with no ScUD criterion.

#### Unidimensionality and Local Independence

To assess the dimensionality of the 9 criteria, a prerequisite to IRT, we fitted a 1-factor model using confirmatory factor analysis (CFA). Analysis was done using Mplus 8 [38]. Unidimensionality was confirmed when the CFA model showed adequate fit by comparative fit index or Tucker-Lewis Index of 0.95 or more and root mean squared error of approximation 0.06 or less [39]. Factor loadings below 0.40 were considered to be weakly related to the underlying construct [40].

We verified local independence between items using standardized z-scores with Mplus 8 [38,41]. Any significant residual correlation between the pairs of items (bivariate), after accounting for the underlying latent trait, would violate the assumption of local independence. Residual correlation between the items is observed if either the standardized *z*-scores for the different combinations of item responses are greater than 1.96 or below –1.96 (corresponding to a *P* value <.05), or if the chi-square value (an overall measure for both items, combining all the possible combinations) is greater than 3.84 (*P*<.05).

#### Item Response Theory

A 2-parameter logistic (2PL) IRT model was performed with the 9 criteria. Our scale was dichotomous and the 2PL model allowed us to examine the difficulty (inversely related to frequency; rarely endorsed criteria are considered more difficult) and discrimination (how well the criterion differentiated between respondents with high and low difficulty of the condition) of each criterion. Item characteristic curves (ICCs) were generated to display the estimated probability of endorsing each criterion across the underlying continuum. In the ICC, the difficulty parameter was the point on the x-axis where the probability of endorsing a criterion was 0.5 (curve toward the right indicates criteria of greater difficulty), and discrimination is the slope of the curve at that point (steeper slopes indicate greater discrimination). We generated item information curves, an indicator on how each item contributes variably to the total test information. Total information curves were generated to show their ability to discriminate individuals along the latent trait severity spectrum [33,40,42].

Description of the sample (mean, SD, and percentage) was performed with JMP; CFA and IRT (psychometric analysis) were performed with Mplus 8 [38].

### Ethics Approval

The survey was anonymous and confidential, and met French regulation ethics standards for noninterventional research after institutional review board (Sanpsy/University of Bordeaux) review [43]. Participation was voluntary with no financial compensation. The questionnaire was distributed with an information note presenting the investigation, consent collection, confidentiality, and legal issues.

## Results

### Sociodemographic Information

Of the 300 participants, 171 were women (57.0%), mean age was 27 years (SD 18.9 years), and 160 were under 18 years (53.3%). The youngest participant was 11 years and the oldest was 84 years. Almost all participants (n=297, 99.0%) reported daily screen use (Table 2).

**Table 2:** Demographic characteristics, screen use (any), and screen use disorder (n=300).

Characteristics		Sample
Age, mean (SD)		27 (18.9)
Age, median		15
Males, n (%)		129 (43.0)
<18-year olds, n (%)		160 (53.3)
Screen use (every day), n (%)		297 (99.0)
**Screen use disorder criteria (cumulative), n (%)**		
	0	166 (55.3)
	≥1	134 (44.7)
	≥2	58 (19.3)
	≥3	23 (7.7)
	≥4	7 (2.3)
	≥5	5 (1.7)
	≥6	3 (1.0)
	7	1 (0.3)

### Screen Use Disorder Diagnosis

Most of the sample reported no criteria (n=166, 55.3%), 134 participants (44.7%) reported at least one criterion (potential problem users), and 5 participants (1.7%) reported 5 criteria or more and qualified for a potential ScUD (Table 2). Adolescents (defined as 11-17 years; mean age 12.92 years, SD 1.50 years) were significantly more likely to endorse at least one ScUD criteria than adults (defined as being aged above 18 years, mean age 43.2 years, SD 16.5 years; 97/300, 32.3% vs 37/300, 12.3%; *P*<.001).

The prevalence of each criterion is reported in Table 3. The most endorsed were *loss of control* (60/300, 20.0%) and *preoccupation* (52/300, 17.3%). The less endorsed were *losing an opportunity* (6/300, 2.0%) and *tolerance* (7/300, 2.3%).

**Table 3:** Parameter estimates from confirmatory factor analysis/Item Response Theory analysis in screen use disorder.

Screen use disorder criteria	Factor loading^a^			Screen use (n=300)			
				Item Response Theory parameters			
	1-factor model	Prevalence (N=300), n (%)	*(b)* Difficulty (SE)		Difficulty rank	*(a)* Discrimination (SE)	*(c)* Discrimination rank
Preoccupation	0.726	52 (17.3)	1.279 (0.224)		1	1.882 (0.618)	2
Withdrawal	0.457	10 (3.3)	3.656 (1.515)		9	1.058 (0.569)	7
Tolerance	0.493	7 (2.3)	3.290 (0.855)		8	1.404 (0.500)	5
Loss of control	0.477	60 (20.0)	1.806 (0.484)		2	0.884 (0.284)	9
Loss of interests	0.779	21 (7.0)	1.962 (0.350)		3	2.027 (0.714)	1
Continue despite problems	0.499	44 (14.7)	2.009 (0.468)		4	1.047 (0.317)	8
Deceive/cover up	0.649	10 (3.3)	2.658 (0.523)		5	1.735 (0.564)	3
Escape adverse mood	0.568	21 (7.0)	2.664 (0.704)		6	1.174 (0.429)	6
Risk/lose relationship/opportunities	0.650	6 (2.0)	3.020 (0.856)		7	1.721 (0.823)	4

^a^Model fit indices: comparative fit index 1.000; Tucker-Lewis Index 1.026; root mean square error of approximation ≤0.0001.

### Dimensionality, Local Independence, and IRT Analysis

**Figure 2:**
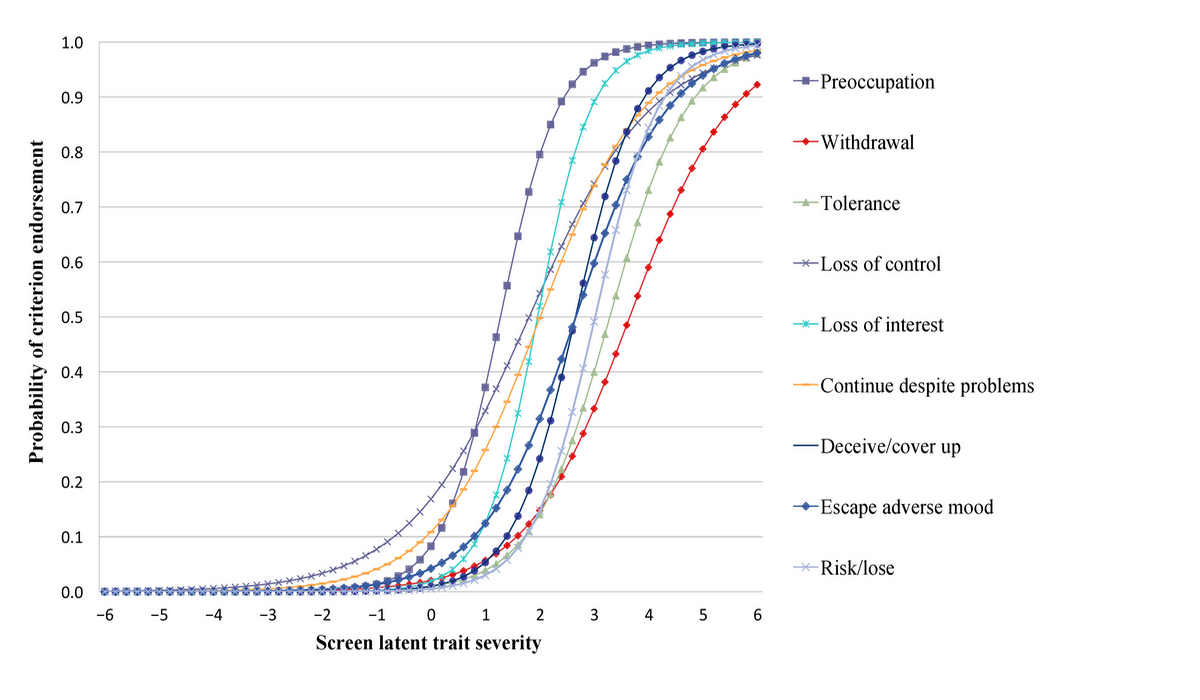
ICC for IGD criteria adapted to screen use disorder in the general population sample of a French suburban city. ICC: item characteristics curve; IGD: internet gaming disorder.

Unidimensionality was confirmed by all fit indices (comparative fit index 1.000; Tucker-Lewis Index 1.026; root mean square error of approximation ≤0.0001; and factor loading ≥0.4 for each criterion). Local independence was confirmed by the absence of residual correlation between the items (minimum and maximum standardized *z*-scores for the different combinations of item responses were equal to –1.042 and 1.129, respectively; maximal chi-square value was 2.008). All criteria had relatively high factor loading except *tolerance* (0.493), *withdrawal* (0.457), and *loss of control* (0.477), but these also remained above the cut-offs sanctioning unidimensionality. Factor loading for *loss of interest* (0.779) was higher than for any other diagnostic criterion, followed by *preoccupation* (0.726). The criterion *preoccupation* (1.279) had the lowest difficulty to be endorsed, followed by *loss of control* and *loss of interest*. Inversely, the *withdrawal* and *tolerance* criteria showed the highest difficulty. Discrimination parameters ranged from 0.884 to 2.027, indicating a good ability to delineate individuals who were higher versus lower to the latent trait (ICC; Figure 2). Both *Loss of interest* (2.027) and *preoccupation* (1.279) criteria showed a higher discrimination, while *loss of control* showed a lower discrimination (0.884) compared with other criteria (Table 3).

**Figure 3:**
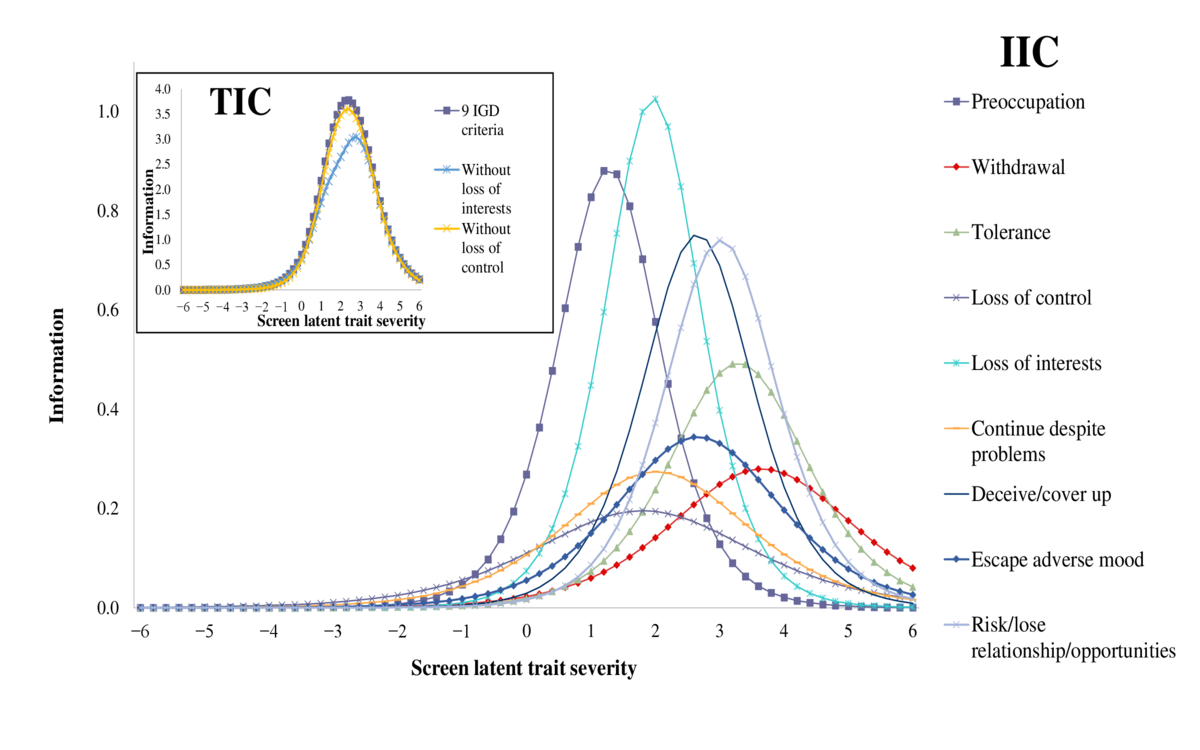
IICs and TICS for IGD criteria adapted to screen use disorder in the general population sample of a French suburban city. IGD: internet gaming disorder; IIC: item information curve; TIC: total information curve.

Item information curves (Figure 3) showed that most discriminating criteria were, in order, *loss of interests*, *preoccupation*, *deceive/cover up,* and *risk/lose relationship/opportunities*, which also provided the most information on the measurement of the latent trait. *Loss of interests* and *preoccupation* criteria also provided the greatest amount of information and high precision across the latent trait severity continuum of ScUD. Loss of control criterion was identified as the least discriminating and the least informative.

Total information curves (Figure 3) showed an increased information across the severity spectrum for the 9 IGD criteria group. Removing the *loss of control* criterion did not seem to affect the ability of the test to capture the disorder phenomenon. However, removing the *loss of interests* criterion changed the amount of severity information provided by the test. The 3 models brought roughly the same range of severity.

### Screen Use and Screen Activities

In univariate analysis, participants with no ScUD criterion were more likely to report *television* (*P*<.001) as the most used screen (Table 4). Participants with at least one ScUD criterion were more likely to have *smartphone* (*P*=.04) and *computer* (*P*=.04) as the most used screens, which they also reported as the most problematic screens: *smartphone* (69/269, 25.7%) and *computer* (61/269, 22.7%). For *tablets* and *handled console* there was no difference between participants with and without at least one ScUD criteria.

Compared with participants with no ScUD criteria, participants with at least one ScUD criteria reported more *video gaming* (*P*<.001) and *communication/social network* (*P*<.001), which they also reported as the most problematic activity: 36.6% (71/194) and 31.4% (61/194), respectively.

In multivariate analysis, when controlled on age and gender, participants with at least one ScUD criterion were more likely to have a *computer* (*P*=.004) as the most used screen type. For activities, they reported more *videogaming* (*P*=.002) and *communication/social network* (*P*=.03) compared with participants with no ScUD criteria. Besides, a new association was found between having at least one ScUD criterion and *watching news and research of information* (*P*=.002) that was not observed in the univariate analysis.

**Table 4:** Main screen and activity for participants with no ScUD^a^ criteria and at least one ScUD criteria. Description of screen type and activities considered as problematic for participants with at least one ScUD criteria.

Activities (several answers possible)		Participants with no ScUD criteria (n=166)	Participants with 1 or more ScUD criteria (n=134)	Univariate analysis: *P* value (Pearson)	Multivariate analysis: adjusted *P* value (logistic regression)
**Screen type (several answers possible), n (%)**					
	TV	103 (62.0)	57 (42.5)	<.001	.06^b^
	Smartphone	55 (33.1)	60 (44.8)	.04	.41
	Computer	24 (14.5)	32 (23.9)	.04	.004
	Tablet	20 (12.0)	23 (17.2)	.21	.73
	Handheld console	7 (4.2)	11 (8.2)	.15	.30
**Screen type reported as problematic (several answers possible; n=269), n (%)**					
	TV	—^c^	58 (21.6)	—	—
	Smartphone	—	69 (25.7)	—	—
	Computer	—	61 (22.7)	—	—
	Tablet	—	45 (16.7)	—	—
	Handheld console	—	31 (11.5)	—	—
	Other	—	5 (1.9)	—	—
**Screen activities reported as problematic (several answers possible; n=194)**					
	News and information	—	17 (8.8)	—	—
	Work-related activities	—	8 (4.1)	—	—
	Others	—	27 (13.9)	—	—
	Communication/social	—	71 (36.6)	—	—
	Video gaming	—	61 (31.4)	—	—
	Purchase	—	6 (3.1)	—	—
	Gambling	—	4 (2.1)	—	—

^a^ScUD: screen use disorder.

^b^Not significant.

^c^Not applicable.

## Discussion

### Principal Findings

This is the first study to combine description of screen use and exploration of the dimensionality and psychometric validity of the 9 IGD DSM-5 criteria adapted to a potential “ScUD” among a general population sample. Prevalence of ScUD was 1.7% (5/300) in our sample. Our results confirm the initial hypothesis of unidimensionality of the 9 IGD DSM-5 criteria adapted to ScUD.

Almost all participants (297/300, 99.0%) of this survey used screens daily, reflecting a high level of equipment use in daily life. ScUD criteria were characterized by the DSM-5 IGD criteria adapted to screen use. The majority of our participants (166/300, 55.3%) self-reported none of the criteria in the past 12 months. However, a notable proportion (134/300, 44.7%) self-reported at least one criterion and a screen type or screen activity as problematic in the past 12 months. This can be interpreted as a need for support and advice for better use of screens in that population. “Screen addiction” prevalence (≥5 criteria endorsed) was 1.7% (5/300), which is in range with the prevalence of IGD (2.0%) in population-based studies [23,44,45]. Two recent meta-analysis on gaming disorder prevalence, a “screen-related addiction,” showed prevalence in the same order of magnitude, 3.0% and 3.3%, respectively [44,45]. Our results are interesting in that they go against lay beliefs of a very high prevalence of “screen addiction.” For those people satisfying 5 or more ScUD criteria, a persistent and recurrent use of screens leading to clinically significant impairment or distress could be assumed, similar to IGD in the DSM-5 [17,18]. In this particular situation, it should be assumed that advice on screen use would be insufficient, and that an addiction-oriented intervention would be useful. There were more adolescents than adults with at least one ScUD criterion (97/300, 32.3% vs 37/300, 12.3%). As many as 2 adults and 3 teenagers met the threshold of 5 criteria for ScUD.

Screens most used differed between participants with at least one ScUD criteria or no criteria. When controlled for age and gender, participants with at least one ScUD criterion were significantly more likely to use computers as the main screen. This may be explained by the activities performed on computers. These participants reported more *video gaming*, *communication/social network*, and *watching news and research of information*, all of which are commonly done on computers. Screens and activities reported as problematic by participants with at least one ScUD criterion were similar to the screens used (eg, smartphone, computers) and activities (eg, video gaming, communication/social network) performed the most, a result that may be of interest for prevention. There was a group of screen users that reported some problem with use and as such is likely to be responsive to interventions focused on related support.

Our study showed unidimensionality of the 9 IGD DSM-5 criteria adapted to ScUD. The model showed adequate fit and the criteria reflected 1 underlying latent trait (ScUD). Moreover, we found no residual correlation between the items, and thus confirmed local independence, a fundamental assumption in IRT models. This means that the items were correlated only through the latent trait that the test is measuring [46]. Some criteria had specific psychometric characteristics. *Loss of interest* (losing interest or reducing participation in other recreational activities) and *preoccupation* (being absorbed by screen use and thinking about it) loaded more strongly than other diagnostic criteria, indicating that they fit well with the 1-factor model, similar to results from a parent-reported survey of screen media “addiction” in children [14]. These criteria were among the more frequently endorsed, and had higher discrimination than others. Thus, both *loss of interest* and *preoccupation* criteria seem to capture the less severe end of the diagnostic spectrum, and the criteria well differentiated between respondents with high and low screen use severity. By identifying participants with less severe ScUD, these items are potentially useful as early indicators of ScUD [47]. It would be interesting to assess, within a prospective cohort of adolescents, whether the occurrence of these criteria predicts a subsequent ScUD.

*Withdrawal* and *tolerance* criteria had the lowest factor loading and showed the highest difficulty and moderate discrimination power, similar to results in a general population study of children [14]. Our results suggest that these criteria may not be relevant to define ScUD. By contrast, in some IGD surveys including population of video gamers with significant gaming time, *withdrawal* and *tolerance* had higher factor loadings and seemed discriminating [19,20], suggesting that very high and regular level of gaming practice may promote tolerance and withdrawal symptoms. Recently, the World Health Organization (WHO) specified its own gaming disorder criteria in the 11th revision of the International Classification of Diseases (ICD-11) [48]. *Tolerance* and *withdrawal* criteria were removed, as well as *preoccupation*, *deceive/cover up*, and *escape adverse mood*. Additional studies among the general population are thus needed to determine to what extent withdrawal and tolerance are related to the intensity of screen use and characterize potential ScUD.

The *loss of control* criterion (feeling that you should use less screens, but being unable to cut back on the amount of time spent watching it) had a lower factor loading, a lower discrimination power, and was among the less difficult (more frequent) criteria. This suggests that this criterion is frequent in a population without ScUD, perhaps due to high overall screen use exposure [1,3]. Including a criterion with poor discrimination may increase the risk for false-positive diagnosis, especially at the lower range of difficulty (high frequency) [47]. In previous studies on IGD [19,20] this criterion had low standard in terms of factor loading, discrimination, and difficulty. However, this result is questionable because this criterion is reported to be a central criterion of addiction [17,49]. By contrast, in another study about screen addiction, *loss of control* showed the highest factor loading in children [14], possibly because reports were from children’s caregivers, and cessation of use is a source of conflict between parents and children. More studies are therefore needed to evaluate the potential importance of this criterion in ScUD.

### Limitations

Study limitations are to be noted. This was a convenience sample with a somewhat low response rate. Survey respondents represented 6.60% (401/6075) of the target population (men and women above 11 years from Martignas-sur-Jalle). Compared with the target population, our final sample was younger (24 years vs 40.5 years), mainly due to a higher proportion of 12-18 year olds (160/300, 53.3%, in our sample vs 784/6075, 12.9%, in the target population). Gender ratio was comparable (3159/6075, 52.0%, women in target population vs 171/300, 57.0%, in our sample). As our questionnaire was self-administered, risk of participant misinterpretation should be considered. However, we used the operationalized formulations for IGD assessment [18]. As a result of missing information, 101 questionnaires (responses) were excluded and there was a higher rate of adolescents among our sample. This could suggest that the questionnaire may have been of little interest to some participants, or might have been difficult to understand, or that adolescents might feel more concerned by this survey. An important element for the validity of the ScUD diagnostic criteria is to determine whether criteria or criteria sets function differently across population subgroups, such as age or sex. As our sample is composed of adolescents and adults, it would be interesting to see whether criteria behave differently according to age. However, in this study, the prevalence of some criteria was too small and thus such an analysis could not be performed here. Additional data in samples more likely to endorse ScUD criteria should be collected and analyzed for differential item functioning. Further studies should investigate the relationships between ScUD items to determine whether there is some local dependence, indicating a possible redundancy. Finally, because craving was not part of IGD criteria [18], no craving criterion was assessed. Some studies suggest that craving should be included [50,51], as it has a high prevalence in samples of those with IGD [52,53], and is the most specific criterion for many substance use disorders [37]. Additional studies should thus be carried out by including craving.

### Conclusions

We described screen use in a French community sample and have shown that the adaptation of the DSM-5 IGD criteria to “ScUD” has good psychometric validity. Endorsement of diagnostic criteria in the past 12 months could be interpreted as current complaints and impairment of the users, strengthening the possibility for ScUD to qualify as a disorder. Further studies are needed to confirm the validity of ScUD diagnosis and its negative consequences. We suggest that there may be similarities between different screen-related addictions, thus allowing for a broader tool to encompass the screen activities. Future studies will have to determine whether the type of screen/screen activity is related to the likelihood of ScUD diagnosis, the validity of a craving criterion, if all criteria are needed or if some should be removed or replaced, and if the diagnosis threshold of 5 is appropriate. Screen use and its consequences represent an important emerging field for addiction research.

## Appendix

Multimedia Appendix 1 English and French versions of the questionnaire.

## Abbreviations

2PL: 2-parameter logistic
CFA: confirmatory factor analysis
DSM-5: Diagnostic and Statistical Manual of Mental Disorders, Fifth Edition
ICD: International Classification of Diseases
IGD: internet gaming disorder
IRT: Item Response Theory
ScUD: screen use disorder
WHO: World Health Organization
