# Supplementary material for: Item Response Theory Analyses of Diagnostic and Statistical Manual of Mental Disorders, Fifth Edition (DSM-5) Criteria Adapted to Screen Use Disorder: Exploratory Survey
Source: J Med Internet Res. 2022 Jul 27;24(7):e31803. doi: 10.2196/31803 (PMC9377463; doi:10.2196/31803)
Supplement: Multimedia Appendix 1 [file jmir_v24i7e31803_app1.pdf]

APPENDIX: Translated and original French versions of the screens use questionnaire

Source: Boudard M, Alexandre JM, Kervran C, Jakubiec L, Shmulewitz D, Hasin D, Fournet L, Rassis C, Claverie P, Serre F, Auriacombe M Item Response Theory Analyses of Diagnostic and Statistical Manual of Mental Disorders, Fifth Edition (DSM-5) Criteria Adapted to Screen Use Disorder: Exploratory Survey J Med Internet Res 2022;24(6):e31803 URL: https://www.jmir.org/2022/6/e31803/ doi: 10.2196/31803

Anonymous survey of screen use
Teenagers and Adults

The city of Martignas and its partners wish to study the place taken by screens and screen uses in Martignas families: televisions, computers, mobile phones, tablets and handheld game consoles. An anonymous and optional survey is conducted by Sanpsy research team UMR 6033 CNRS /University of Bordeaux and the Addictology Department of the Charles Perrens Hospital Center - Bordeaux University Hospital.
Could you give us a few minutes of your time and answer these few questions? Thank you.

You are: a Man □; a Woman □
Please specify your age (*indicate the number*): ______ year old

Part 1: Television

Do you own one/several television(s)? *(Check box)* Yes □ ; No □
If yes, how many? (*Indicate the number)* ______
If no, please proceed to the next part

How often have you used it in the past 30 days? *(Check only one box)*
Every day or almost every day □; More than one day out of two □; Less than one day out of two; Never or Almost never □

How long do you estimate using it per day? *(Check only one box)*
Less than 1 hour □; between 1 and 2 hours □; between 2 and 3 hours □; between 3 and 5 hours □ ; more than 5 hours □

At what time(s) of the day? *(Check one or several boxes)*
Morning □ ; Afternoon □ ; Evening ; During meals □ ; At bedtime □ ; Night □

How do you use it most of the time? *(Check only one box)*
Alone □ ; With my family □ ; With my friends □

What main use(s) do you have on your own*? (Check one or several boxes)*
Work □ ; Education or Training □ ; News □ ; Documentation □ ; Teleshopping □ ; Video games □ ; Gambling / Sports betting □ ; Other entertainment(s) □

What main use(s) do you have as a group (family, friends…)? *(Check one or several boxes)*
Work □ ; Education or Training □ ; News □ ; Documentation □ ; Teleshopping □ ; Video games □ ; Gambling / Sports betting □ ; Other entertainment(s) □

Part 2: Computer

Do you own one/several computer(s)? *(Check box)* Yes □ ; No □
If yes, how many? (*Indicate the number)* ______
If no, please proceed to the next part

How often have you used it in the past 30 days? *(Check only one box)*
Every day or almost every day □; More than one day out of two □; Less than one day out of two; Never or Almost never □

How long do you estimate using it per day? *(Check only one box)*
Less than 1 hour □; between 1 and 2 hours □; between 2 and 3 hours □; between 3 and 5 hours □ ; more than 5 hours □

At what time(s) of the day? *(Check one or several boxes)*
Morning □ ; Afternoon □ ; Evening ; During meals □ ; At bedtime □ ; Night □

How do you use it most of the time? *(Check only one box)*
Alone □ ; With my family □ ; With my friends □; At work □

What main use(s) do you have on your own? *(Check one or several boxes)*
Communication (email, skype, forums…) □; Social networks □ ; Work □ ; Education or Training □ ; News □ ; Search for information on the Internet □; Other form of documentation □; Administrative procedures □ ; Purchases □ ; Video games □ ; Gambling / Sports betting □ ; Other entertainment(s) □

What main use(s) do you have as a group (family, friends…)? *(Check one or several boxes)*
Communication (email, skype, forums…) □; Social networks □ ; Work □ ; Education or Training □ ; News □ ; Search for information on the Internet □; Other form of documentation □; Administrative procedures □ ; Purchases □ ; Video games □ ; Gambling / Sports betting □ ; Other entertainment(s) □

Part 3: Smartphone

Do you own one/several smartphone(s)? *(Check box)* Yes □ ; No □
If yes, how many? (*Indicate the number)* _____
If no, please proceed to the next part

How often have you used it in the past 30 days? *(Check only one box)*
Every day or almost every day □; More than one day out of two □; Less than one day out of two; Never or Almost never □

How long do you estimate using it per day? *(Check only one box)*
Less than 1 hour □; between 1 and 2 hours □; between 2 and 3 hours □; between 3 and 5 hours □ ; more than 5 hours □

At what time(s) of the day? *(Check one or several boxes)*
Morning □ ; Afternoon □ ; Evening ; During meals □ ; At bedtime □ ; Night □

How did you learn to use it?
Alone □ ; With a seller □ ; With family □ ; With friend(s) □; on the Internet □ ; Other □

What main use(s) do you have on your own? *(Check one or several boxes)*
Communication (email, skype, forums…) □; Social networks □ ; Work □ ; Education or Training □ ; News □ ; Search for information on the Internet □; Other form of documentation □; Administrative procedures □ ; Purchases □ ; Video games □ ; Gambling / Sports betting □ ; Other entertainment(s) □

What main use(s) do you have as a group (family, friends…)? *(Check one or several boxes)*
Communication (email, skype, forums…) □; Social networks □ ; Work □ ; Education or Training □ ; News □ ; Search for information on the Internet □; Other form of documentation □; Administrative procedures □ ; Purchases □ ; Video games □ ; Gambling / Sports betting □ ; Other entertainment(s) □

Part 4: Tablet

Do you own one/several tablet(s)? *(Check box)* Yes □ ; No □
If yes, how many? (*Indicate the number)* _____
If no, please proceed to the next part

How often have you used it in the past 30 days? *(Check only one box)*
Every day or almost every day □; More than one day out of two □; Less than one day out of two; Never or

Almost never □

How long do you estimate using it per day? *(Check only one box)*
Less than 1 hour □; between 1 and 2 hours □; between 2 and 3 hours □; between 3 and 5 hours □ ; more than 5 hours □

At what time(s) of the day? *(Check one or several boxes)*
Morning □ ; Afternoon □ ; Evening ; During meals □ ; At bedtime □ ; Night □

How do you use it most of the time? *(Check only one box)*
Alone □ ; With my family □ ; With my friends □; At work □

What main use(s) do you have on your <u>own</u>? *(Check one or several boxes)*
Communication (email, skype, forums…) □; Social networks □ ; Work □ ; Education or Training □ ; News □ ; Search for information on the Internet □; Other form of documentation □; Administrative procedures □ ; Purchases □ ; Video games □ ; Gambling / Sports betting □ ; Other entertainment(s) □

What main use(s) do you have as <u>a group (family, friends…)?</u> *(Check one or several boxes)*
Communication (email, skype, forums…) □; Social networks □ ; Work □ ; Education or Training □ ; News □ ; Search for information on the Internet □; Other form of documentation □; Administrative procedures □ ; Purchases □ ; Video games □ ; Gambling / Sports betting □ ; Other entertainment(s) □

Part 5: Handheld game console

Do you own one/several handheld console(s)? *(Check box)* Yes □ ; No □
If yes, how many? (*Indicate the number)* _____
<u>If no, please proceed to the next part</u>

How often have you used it in the past 30 days? *(Check only one box)*
Every day or almost every day □; More than one day out of two □; Less than one day out of two; Never or Almost never □

How long do you estimate using it per day? *(Check only one box)*
Less than 1 hour □; between 1 and 2 hours □; between 2 and 3 hours □; between 3 and 5 hours □ ; more than 5 hours □

At what time(s) of the day? *(Check one or several boxes)*
Morning □ ; Afternoon □ ; Evening ; During meals □ ; At bedtime □ ; Night □

How do you use it most of the time? *(Check only one box)*
Alone □ ; in group or online: With my family □ ; With friends □

In the last 12 months *(Check appropriate boxes)***:**

* Did you spend a lot of time thinking about screens even when you were not using them, or planning when you could use them next? □
* Did you feel restless, irritable, moody, angry, anxious or sad when attempting to cut down or stop using screens, or when you were unable to use screens? □
* Did you feel the need to use screens for increasing amounts of time, use more exciting screens, or use more powerful equipment to get the same amount of excitement you used to get? □
* Did you feel that you should use screens less, but were unable to cut back on the amount of time you spend watching screens? □
* Did you lose interests in or reduce participation in other recreational activities (hobbies, meetings with friends) due to screens? □
* Did you continue to use screens even though you were aware of negative consequences for you? □
If you checked previous box, which kind(s) of problem(s)?
not getting enough sleep / restless sleep □

being late to school/work □
spending too much money □
having arguments with others □
neglecting important duties □
weight problems □
eyesight problems □
other problems □, please specify: ____

* Did you lie to family, friends or others about how much you watch screens, or try to keep your family or friends from knowing how much you use screens? □
* Did you use screens to escape from or forget about personal problems, or to relieve uncomfortable feelings such as guilt, anxiety, helplessness or depression? □
* Did you risk or lose significant relationships, or job, educational or career opportunities because of screens? □

If you checked one or more boxes, which type(s) of screen(s) are involved? *(Check one or several boxes)*
Television □ ; Computer □ ; Smartphone □ ; Tablet □ ; Handheld console □ ;
Another screen □, please specify: _______________

If you checked one or more boxes, which type(s) of use(s) are involved? *(Check one or several boxes)*
Communication (email, skype, forums…) □; Social networks □ ; Work □ ; News □ ; Search for information on the Internet □; Other form of documentation □; Purchases □ ; Video games □ ; Gambling / Sports betting □ ; Other entertainment(s) □

Enquête anonyme sur les usages des écrans
Adolescents et adultes

Source: Boudard M, Alexandre JM, Kervran C, Jakubiec L, Shmulewitz D, Hasin D, Fournet L, Rassis C, Claverie P, Serre F, Auriacombe M Item Response Theory Analyses of Diagnostic and Statistical Manual of Mental Disorders, Fifth Edition (DSM-5) Criteria Adapted to Screen Use Disorder: Exploratory Survey J Med Internet Res 2022;24(6):e31803 URL: https://www.jmir.org/2022/6/e31803/ doi: 10.2196/31803

La ville de Martignas et ses partenaires souhaitent étudier la place prise par les écrans et les usages dans les familles martignassaises : télévisions, ordinateurs, téléphones portables, tablettes et consoles de jeu portables. Une enquête **anonyme et facultative** est menée par l'équipe de recherche Sanpsy – UMR 6033 CNRS/Université de Bordeaux et le Pôle Addictologie du Centre Hospitalier Charles Perrens – CHU de Bordeaux.
Pourriez-vous nous accorder quelques minutes et répondre à ces quelques questions ?
Nous vous remercions.

Vous êtes : un Homme ☐ ; une Femme ☐
Merci de préciser votre âge (*Indiquez le chiffre*) : _____ ans

Partie 1 : Télévision

Possédez-vous un/plusieurs téléviseur(s) ? *(Cochez la case)* Oui ☐ ; Non ☐
Si oui, combien ? (*Indiquez le chiffre*) _____
Si non, merci de passer au cadre suivant

A quelle fréquence l'avez-vous utilisé dans les 30 derniers jours ? *(Cochez une seule case)*
Tous les jours ou presque ☐ ; Plus d'un jour sur deux ☐ ; Moins d'un jour sur deux ☐ ; Jamais ou presque ☐

Combien de temps estimez-vous l'utiliser par jour ? *(Cochez une seule case)*
Moins de 1 heure ☐ ; entre 1 et 2 heures ☐ ; entre 2 et 3 heures ☐ ; entre 3 et 5 heures ☐ ; plus de 5 heures ☐

A quel(s) moment(s) de la journée ? *(Cochez une ou plusieurs cases)*
Matin ☐ ; Après-midi ☐ ; Soir ☐ ; Pendant les repas ☐ ; Au coucher ☐ ; Nuit ☐

Comment l'utilisez-vous la plupart du temps ? *(Cochez une seule case)*
Seul ☐ ; Avec ma famille ☐ ; Avec mes amis ☐

Quel(s) usage(s) principal(aux) en avez-vous seul ? *(Cochez une ou plusieurs cases)*
Travail ☐ ; Education ou Formation ☐ ; Actualités ☐ ; Documentation ☐ ; télé-achats ☐ ; Jeux vidéo ☐ ;
Jeux de hasard et d'argent / Paris sportifs ☐ ; Autre(s) divertissement(s) ☐

Quel(s) usage(s) principal(aux) en avez-vous en groupe (famille ou amis) ? *(Cochez une ou plusieurs cases)*
Travail ☐ ; Education ou Formation ☐ ; Actualités ☐ ; Documentation ☐ ; télé-achats ☐ ; Jeux vidéo ☐ ;
Jeux de hasard et d'argent / Paris sportifs ☐ ; Autre(s) divertissement(s) ☐

Partie 2 : Ordinateur

Possédez-vous un/plusieurs ordinateur(s) ? *(Cochez la case)* Oui ☐ ; Non ☐
Si oui, combien ? (*Indiquez le chiffre*) _____
Si non, merci de passer au cadre suivant

A quelle fréquence l'avez-vous utilisé dans les 30 derniers jours ? *(Cochez une seule case)*
Tous les jours ou presque ☐ ; Plus d'un jour sur deux ☐ ; Moins d'un jour sur deux ☐ ; Jamais ou presque ☐

Combien de temps estimez-vous l'utiliser par jour ? *(Cochez une seule case)*
Moins de 1 heure ☐ ; entre 1 et 2 heures ☐ ; entre 2 et 3 heures ☐ ; entre 3 et 5 heures ☐ ; plus de 5 heures ☐

A quel moment de la journée ? *(Cochez une ou plusieurs cases)*

Matin ☐ ; Après-midi ☐ ; Soir ☐ ; Pendant les repas ☐ ; Au coucher ☐ ; Nuit ☐

Comment l'utilisez-vous la plupart du temps ? *(Cochez une seule case)*
Seul ☐ ; Avec ma famille ☐ ; Avec mes amis ☐ ; Au travail ☐

Quel(s) usage(s) principal(aux) en avez-vous seul ? *(Cochez une ou plusieurs cases)*
Communication (messagerie, skype, forums) ☐ ; Réseaux sociaux ☐ ; Travail ☐ ; Education ou Formation ☐ ; Actualités ☐ ; Recherche d'informations sur Internet ☐ ; Autre forme de Documentation ☐ ; Administratif (contact banque, CAF, Pôle Emploi…) ☐ ; Achats ☐ ; Jeux vidéo ☐ ; Jeux de hasard et d'argent / Paris sportifs ☐ ; Autre(s) divertissement(s) ☐

Quel(s) usage(s) principal(aux) en avez-vous en groupe (famille ou amis) ? *(Cochez une ou plusieurs cases)*
Communication (messagerie, skype, forums) ☐ ; Réseaux sociaux ☐ ; Travail ☐ ; Education ou Formation ☐ ; Actualités ☐ ; Recherche d'informations sur Internet ☐ ; autre forme de documentation ☐ ; Administratif (contact banque, CAF, Pôle Emploi…) ☐ ; Achats ☐ ; Jeux vidéo ☐ ; Jeux de hasard et d'argent / Paris sportifs ☐ ; Autre(s) divertissement(s) ☐

Partie 3 : Smartphone

Possédez-vous une/plusieurs smartphone(s) ? *(Cochez la case)* Oui ☐ ; Non ☐
Si oui, combien ? (*Indiquez le chiffre*) _____
Si non, merci de passer au cadre suivant

A quelle fréquence l'avez-vous utilisé dans les 30 derniers jours ? *(Cochez une seule case)*
Tous les jours ou presque ☐ ; Plus d'un jour sur deux ☐ ; Moins d'un jour sur deux ☐ ; Jamais ou presque ☐

Combien de temps estimez-vous l'utiliser par jour ? *(Cochez une seule case)*
Moins de 1 heure ☐ ; entre 1 et 2 heures ☐ ; entre 2 et 3 heures ☐ ; entre 3 et 5 heures ☐ ; plus de 5 heures ☐

A quel moment de la journée ? *(Cochez une ou plusieurs cases)*
Matin ☐ ; Après-midi ☐ ; Soir ☐ ; Pendant les repas ☐ ; Au coucher ☐ ; Nuit ☐

Comment l'utilisez-vous la plupart du temps ? *(Cochez une seule case)*
Seul ☐ ; Avec ma famille ☐ ; Avec mes amis ☐ ; Au travail ☐

Quel(s) usage(s) principal(aux) en avez-vous seul ? *(Cochez une ou plusieurs cases)*
Communication (messagerie, skype, forums) ☐ ; Réseaux sociaux ☐ ; Travail ☐ ; Education ou Formation ☐ ; Actualités ☐ ; Recherche d'informations sur Internet ☐ ; Autre forme de documentation ☐ ; Administratif (contact banque, CAF, Pôle Emploi…) ☐ ; Achats ☐ ; Jeux vidéo ☐ ; Jeux de hasard et d'argent / Paris sportifs ☐ ; Autre(s) divertissement(s) ☐

Quel(s) usage(s) principal(aux) en avez-vous en groupe (famille ou amis) ? *(Cochez une ou plusieurs cases)*
Communication (messagerie, skype, forums) ☐ ; Réseaux sociaux ☐ ; Travail ☐ ; Education ou Formation ☐ ; Recherche d'informations sur Internet ☐ ; Autre forme de documentation ☐ ; Administratif (contact banque, CAF, Pôle Emploi…) ☐ ; Achats ☐ ; Jeux vidéo ☐ ; Jeux de hasard et d'argent / Paris sportifs ☐ ; Autre(s) divertissement(s) ☐

Partie 4 : Tablette

Possédez-vous une/plusieurs tablette(s) ? *(Cochez la case)* Oui ☐ ; Non ☐
Si oui, combien ? (*Indiquez le chiffre*) _____
Si non, merci de passer au cadre suivant

A quelle fréquence l'avez-vous utilisée dans les 30 derniers jours ? *(Cochez une seule case)*
Tous les jours ou presque ☐ ; Plus d'un jour sur deux ☐ ; Moins d'un jour sur deux ☐ ; Jamais ou presque ☐

Combien de temps estimez-vous l'utiliser par jour ? *(Cochez une seule case)*

Moins de 1 heure ☐ ; entre 1 et 2 heures ☐ ; entre 2 et 3 heures ☐ ; entre 3 et 5 heures ☐ ; plus de 5 heures ☐

A quel moment de la journée ? *(Cochez une ou plusieurs cases)*
Matin ☐ ; Après-midi ☐ ; Soir ☐ ; Pendant les repas ☐ ; Au coucher ☐ ; Nuit ☐

Comment l'utilisez-vous la plupart du temps ? *(Cochez une seule case)*
Seul ☐ ; Avec ma famille ☐ ; Avec mes amis ☐ ; Au travail ☐

Quel(s) usage(s) principal(aux) en avez-vous seul ? *(Cochez une ou plusieurs cases)*
Communication (messagerie, skype, forums) ☐ ; Réseaux sociaux ☐ ; Travail ☐ ; Education ou Formation ☐ ; Actualités ☐ ; Recherche d'informations sur Internet ☐ ; Autre forme de documentation ☐ ; Administratif (contact banque, CAF, Pôle Emploi…) ☐ ; Achats ☐ ; Jeux vidéo ☐ ; Jeux de hasard et d'argent / Paris sportifs ☐ ; Autre(s) divertissement(s) ☐

Quel(s) usage(s) principal(aux) en avez-vous en groupe (famille ou amis) ? *(Cochez une ou plusieurs cases)*
Communication (messagerie, skype, forums) ☐ ; Réseaux sociaux ☐ ; Travail ☐ ; Education ou Formation ☐ ; Actualités ☐ ; Recherche d'informations sur Internet ☐ ; Autre forme de Documentation ☐ ; Administratif (contact banque, CAF, Pôle Emploi…) ☐ ; Achats ☐ ; Jeux vidéo ☐ ; Jeux de hasard et d'argent / Paris sportifs ☐ ; Autre(s) divertissement(s) ☐

Partie 5 : Console de jeux portable

Possédez-vous une/plusieurs console(s) de jeu portable(s) ? *(Cochez la case)* Oui ☐ ; Non ☐
Si oui, combien ? (*Indiquez le chiffre*) _____
Si non, merci de passer au cadre suivant

A quelle fréquence l'avez-vous utilisée dans les 30 derniers jours ? *(Cochez une seule case)*
Tous les jours ou presque ☐ ; Plus d'un jour sur deux ☐ ; Moins d'un jour sur deux ☐ ; Jamais ou presque ☐

Combien de temps estimez-vous l'utiliser par jour ? *(Cochez une seule case)*
Moins de 1 heure ☐ ; entre 1 et 2 heures ☐ ; entre 2 et 3 heures ☐ ; entre 3 et 5 heures ☐ ; plus de 5 heures ☐

A quel moment de la journée ? *(Cochez une ou plusieurs cases)*
Matin ☐ ; Après-midi ☐ ; Soir ☐ ; Pendant les repas ☐ ; Au coucher ☐ ; Nuit ☐

Comment l'utilisez-vous la plupart du temps ? *(Cochez une seule case)*
Seul ☐ ; A plusieurs ou en réseau : Avec ma famille ☐ / Avec mes amis ☐

Au cours des 12 derniers mois *(cochez la ou les case(s) approprié(es))* :

* Passez-vous beaucoup de temps à penser aux écrans, y compris quand vous n'en utilisez pas, ou à prévoir quand vous pourrez en utiliser à nouveau ? ☐
* Lorsque vous tentez d'utiliser moins d'écrans ou de ne plus en utiliser, ou lorsque vous n'êtes pas en mesure d'utiliser d'écran, vous sentez-vous agité, irritable, d'humeur changeante, anxieux ou triste ? ☐
* Ressentez-vous le besoin d'utiliser des écrans plus longtemps, d'utiliser des écrans plus excitants ou d'utiliser du matériel informatique plus puissant pour atteindre le même état d'excitation qu'auparavant ? ☐
* Avez-vous l'impression que vous devriez utiliser moins d'écrans, mais que vous n'arrivez pas à réduire votre temps d'écran ? ☐
* Avez-vous perdu l'intérêt ou réduit votre participation à d'autres activités (temps pour vos divertissements, amis) à cause des écrans ? ☐
* Avez-vous continué à utiliser des écrans, tout en sachant que cela entraînait chez vous des problèmes ? ☐
Si vous avez coché la case précédente : quel(s) type(s) de problème(s) ?
- Ne pas dormir assez ou mal dormir ☐
- Être en retard à l'école/au travail ☐
- Dépenser trop d'argent ☐
- Se disputer ☐
- Négliger des choses importantes à faire ☐
- Problèmes de poids ☐

- Problèmes de vue ☐
- Autres problèmes, précisez : ____

* Vous arrive-t-il de cacher aux autres, votre famille, vos amis, à quel point vous utilisez des écrans, ou de leur mentir à propos de vos habitudes d'écrans ? ☐

* Avez-vous utilisé des écrans pour échapper à des problèmes personnels ou pour soulager une **humeur** indésirable (exemples : sentiments d'impuissance, de culpabilité, d'anxiété ou de dépression) ☐

* Avez-vous mis en danger ou perdu une relation affective importante, un travail, un emploi ou des possibilités d'études à cause des écrans ? ☐

Si vous avez coché une ou plusieurs cases, quel(s) type d'écran(s) sont en cause ? *(Cochez une ou plusieurs cases)*
Télévision ☐ ; Ordinateur ☐ ; Smartphone ☐ ; Tablette ☐ ; Console de jeux portable ☐ ;
Autre écran ☐ *Précisez* : ______________

Si vous avez coché une ou plusieurs cases, quel(s) type(s) d'usage(s) pose(nt) particulièrement problème ? *(Cochez une ou plusieurs cases)*
Communication (messagerie, skype, forums) ☐ ; Réseaux sociaux ☐ ; Travail ☐ ; Recherche d'informations sur Internet ☐ ; Autre forme de documentation ☐ ; Achats ☐ ; Jeux vidéo ☐ ; Jeux de hasard et d'argent / Paris sportifs ☐ ; Autre(s) divertissements ☐
